# Seroprevalence of hepatitis B virus among people screened at a primary care hospital in Bamenda: a cross-sectional study

**DOI:** 10.11604/pamj.2022.41.237.25728

**Published:** 2022-03-22

**Authors:** Cyprine Neba Funeh, Peter Vanes Ebasone, Eric Mbah Chunga, Fonyuy Nkwawir, Rogers Ajeh, Blaise Barche, Ignatius Fonyong Tebid

**Affiliations:** 1 Abii Specialist Medical Foundation, Bamenda Cameroon,; 2 Clinical Research Education Networking and Consultancy, Yaounde, Cameroon,; 3 Bamenda Regional Hospital, Bamenda, Cameroon

**Keywords:** Prevalence, seroprevalence, hepatitis B virus, HbsAg, Cameroon

## Abstract

**Introduction:**

about 257 million people are infected with hepatitis B virus (HBV) worldwide and the infection is endemic in Africa. The general population HBV seroprevalence remains under-reported in Cameroon.

**Methods:**

this was a cross-sectional study including, 1208 consenting adults selected through consecutive sampling, from April 2015 to November 2018, in the Bamenda Health District. Participants´ demographic data were collected and their blood samples were drawn and tested for hepatitis B Surface Antigen (HBsAg). Data were analysed using SPSS version 24 and Chi-squared and Fisher´s exact tests were used to assess bivariate associations.

**Results:**

the participants´ mean age (years) was 35.9±11.8, and the majority were females 720 (59.6%). The seroprevalence of HBV infection was 5.8% (95% CI: 4.5-7.3), and was significantly higher in males 8.4% (95% CI: 6.2-11.1), p=0.001, age group 30-39 years 8.4% (95% CI: 5.8-11.6), p=0.007 and the Mankon health area (12.7%; 95% CI: 9.1-17.1), p=0.026.

**Conclusion:**

the results suggest that HBV infection could be intermediately endemic in Bamenda, with a higher burden in males, people in their third decade and those from the Mankon health area. This study further underscores a need for extensive screening and vaccination campaigns in Cameroon.

## Introduction

Hepatitis B Virus infection is a global public health problem, especially in developing countries. An estimated 257 million people are living with chronic hepatitis B (defined as HBsAg positive) worldwide, with over 887,000 deaths occurring from hepatitis B related complications such as liver cirrhosis and hepatocellular carcinoma [1].

The hepatitis B virus is commonly transmitted via body fluids such as blood, semen, and vaginal secretions. This can happen through sexual contact, sharing needles, syringes, other drug-injection equipment, or from mother to baby at birth [2]. According to Olayinka *et al*. in 2016, the commonest risk factors for hepatitis B were uvulectomy, presence of tribal marks, sharing of sharp objects and circumcision [3]. The infection can be acute or chronic and the course of the infection may range from mild asymptomatic disease to severe or fulminant hepatitis. Approximately 65 million of hepatitis B infected individuals live in Africa, thus Africa with approximately 17% of the world´s population carries 23% of the burden for HBV infections [4]. In areas of high endemicity where at least 8% are chronic carriers, hepatitis B is mostly contracted during early childhood [5]. Perinatal transmission from an infected mother to her baby is common. About 90% of those infected during the prenatal period, 30% of those infected in early childhood, and 6% of those infected after 5 years of age develop chronic infection [6].

In Cameroon, the prevalence ranges from 6-16% [7, 8]. A study by Frambo *et al*. 2014, in the Buea Health District, reported a 9.7% prevalence of HBsAg among pregnant women, and also noted that lack of awareness of the disease led to a delay in diagnosis and spread of the virus [7]. Recent studies recorded the prevalence as high as 10.7% and 12.14% for HBV among blood donors in hospital blood banks in Cameroon [9, 10].

The prevalence of HBV in suburban populations in Cameroon remains largely underreported and thus this study is carried out to determine the prevalence and distribution of HBV infection in an apparently healthy population who serve as an important source for new infections and identify subpopulations at risk who would benefit from vaccination, while providing baseline data for future assessment of the impact of HBV infection and vaccination.

## Methods

**Study design and setting:** this was a cross-sectional study, carried out in the Bamenda Health District from April 2015 to November 2018. Bamenda is the capital city of the Northwest Region of Cameroon. It is the largest English-speaking city in Cameroon with a population projected at 1,323,716 inhabitants in 2020 [11]. It is a cosmopolitan city with an amalgamation of 6 suburban areas; Mankon, Mendakwe, Nkwen, Chomba, Mbatu and Nsongwa. However, considerable portions of outlying suburban areas, including Bambui, Bafut, Bambili and Akum may be considered as part of the Bamenda greater cosmopolitan area, since urban development initiatives are fast engulfing the said areas. The Bamenda Health District is made up of 16 health areas: Mankon, Alakuma, Azire, Mulang, Nkwen Baptist, Nkwen Urban, Alabukam, Bamendankwe, Ntankah, Atuakom, Alamandom, Akumlam, Nkwen Rural, Ntamba, Mbatchongwe and Ndza.

**Study population, sample size and recruitment procedures:** participants were recruited in the study through a consecutive sampling strategy as they showed up voluntarily in the study facility and consented to undertake free HBV testing. The recruitment was preceded by a series of repeated public announcements over local radio stations and churches in Bamenda Health District, of a free Hepatitis B screening campaign at the Abii Specialist Medical Foundation Hospital at Ngomgham, Mulang health area. Based on a pooled seroprevalence (11.2%) of hepatitis B in Cameroon reported by Bigna JJ *et al*. the minimum sample size for statistical significance at 95% CI was estimated at 153 participants.

The study community was sensitized about HBV, modes of transmission, signs and symptoms, complications for positive individuals, treatment and vaccination. They were then informed of the study. Those above 21-years-old who offered their informed consent were then enrolled by the study investigators. Patient privacy and confidentiality was ensured throughout. We excluded people with a known history of hepatitis B infection. Sociodemographic data (age, sex, occupation and address) were collected using a short-pretested questionnaire. A provisionary vaccination programme was offered to participants who tested negative to prevent HBV infection in future.

**Blood sample collection and HBV testing procedures:** about 2-3ml of venous blood was collected from each participant, these samples were centrifuged at 3000rpm for 5 minutes at room temperature. The serum was used to test for HBsAg using the test strip. We used lateral flow immuno-chromatographic method as a screening test. The DiaSpot® HBsAg rapid test (manufactured by Diaspora Diagnostics, USA) was used for screening samples. Two lab technicians were trained on blood sample collection and standard operating procedures for testing, as enclosed in the test kit package provided by the manufacturer. A positive result meant HBV infectivity, and a negative result indicated the absence of the HBV. The test kits were CE (European Conformity) marked and FDA (Food and Drug Administration) approved.

**Statistical analysis:** we entered data into a Microsoft Excel spreadsheet and imported to SPSS version 24 for MacOs for analysis. Continuous variables were presented as means and standard deviations, while categorical variables were presented as proportions. Simple illustrations like tables, measures of central tendencies and dispersion were obtained for the variables for analysis. Bivariate associations were assessed using Chi-square and Fisher´s exact (when an expected value is <5) tests for categorical variables at P-value <0.05 at 95% confidence interval (CI).

## Results

**Participant characteristics:** overall, 1208 participants took part in the study. The mean age was 35.9±11.8 years, ranging between 21 - 80 years. Most participants were between the ages of 21-29 years 450 (37.3%), 30-39 years 359 (29.7%) and 40-49 years 219 (18.1%). The majority of participants were females, 720 (59.6%). Students and teachers comprised the largest proportion of study participants, 263 (21.8%) and 247 (20.4%) respectively. Regarding the health area of origin, most participants were from Mankon health area 267 (22.1%) and Alakuma 152 (12.6%) (Table 1).

**Table 1:** distribution of age groups, occupation and health areas stratified by sex

Variable	Total, n (%)	Female, n (%)	Male, n (%)
**Age Group**			
21-29	450 (37.3%)	302 (41.9%)	148 (30.3%)
30-39	359 (29.7%)	197 (27.4%)	162 (33.2%)
40-49	219 (18.1%)	124 (17.2%)	95 (19.5%)
50-59	121 (10.0%)	70 (9.7%)	51 (10.5%)
≥60	59 (4.9%)	27 (3.8%)	32 (6.6%)
**Occupation**			
Teacher	263(21.8%)	179(24.9%)	84(17.2%)
Student	247(20.4%)	170(23.6%)	77(15.8%)
Trader	238(19.7%)	108(15.0%)	130(26.6%)
Health Personnel	94(7.8%)	68(9.4%)	21(4.3 %)
Applicant	89(7.4%)	59(8.2 %)	7(1.4%)
Technician	66(5.5%)	6(0.8 %)	49(10.0%)
Accountant	55(4.6%)	32(4.4 %)	20(4.1%)
Driver	52(4.3%)	1(0.1 %)	29(5.9%)
Farmer	30(2.5%)	22(3.1%)	7(1.4%)
Retired	29(2.4%)	14(1.9 %)	14(2.9%)
Hair Dresser	28(2.3%)	16(2.2%)	1(0.2%)
Others	17(1.4%)	45(6.3%)	49(10.0%)
**Health Areas**			
Mankon	267(22.1%)	156(21.7%)	111(22.7%)
Alakuma	152(12.6%)	90(12.5%)	62(12.7%)
Azire	106(8.8%)	65(9.0%)	41(8.4%)
Mulang	99(8.2%)	54(7.5%)	45(9.2%)
Nkwen Baptist	98(8.1%)	53(7.4%)	45(9.2%)
Nkwen Urban	95(7.9%)	58(8.1%)	37(7.6%)
Alabukam	91(7.5%)	66(9.2%)	25(5.1%)
Bamendankwe	78(6.5%)	49(6.8%)	29(5.9%)
Ntankah	66(5.5%)	38(5.3%)	28(5.7%)
Atuakom	42(3.5%)	28(3.9%)	14(2.9%)
Alamandum	38(3.1%)	22(3.1%)	16(3.3%)
Akumlam	38(3.1)	23(3.2%)	15(3.1%)
Nkwen Rural	19(1.6%)	9(1.3%)	10(2.0%)
Ntamba	9(0.7%)	4(0.6%)	5(1.0%)
Mbatchongwe	7(0.6%)	5(0.7%)	2(0.4%)
Ndza	3(0.25)	0(0.0%)	3(0.6%)

**Seroprevalence of hepatitis B:** the seroprevalence of HBV in Bamenda was (5.8%; 95% CI: 4.5 - 7.3). The seroprevalence was significantly higher in males (8.4%; 95% CI: 6.2 - 11.1) compared to females (4.0%; 95% CI: 2.8 - 5.7). People aged 30-39 years recorded the largest proportion of HBV positive cases (8.4%; 95% CI: 5.8-11.6), followed by those 21-29 years scoring (6.4%; 95% CI: 4.5-9.0). HBV was most prevalent among drivers (16.7%; 95% CI: 6.7 - 32.7). The prevalence was highest in the Mankon health area (12.7%; 95% CI: 9.1 - 17.1), followed by Nkwen Baptist (5.1%; 95% CI: 2.0 - 10.8) and Mulang (5.1%; 95% CI: 2.0 - 10.7). The difference in seroprevalence of HBV was significant for sex (p = 0.001), age group (p = 0.007) and health area of origin (p = 0.026) but insignificant for occupation (p = 0.191) (Table 2).

**Table 2:** sero-prevalence of HBsAg by sex, age groups, occupation and health area

Variable	Total	HBsAg Positive, n (%)	95% CI	p-value
**Sex**				
Female	720	29 (4.0%)	2.8-5.7	0.001
Male	488	41 (8.4%)	6.2-11.1	
**Age group**				
21-29	450	29 (6.4%)	4.5-9.0	0.007
30-39	359	30(8.4%)	5.8**-**11.6	
40-49	219	9(4.1%)	2.1-7.4	
50-59	121	2(1.7%)	0.3-5.2	
≥60	59	0(0.0%)	-	
**Occupation**				
Teacher	263	16 (6.1%)	3.7-9.5	0.344
Student	247	11(4.5%)	2.4- 7.6	
Trader	238	16(6.7%)	4.1-10.4	
Health Personnel	89	6(6.7%)	2.9-13.4	
Applicant	66	1(1.5%)	0.2-6.9	
Technician	55	4(7.3%)	2.5-16.4	
Accountant	52	2(3.8%)	0.8-11.8	
Driver	30	5(16.7%)	6.7-32.7	
Farmer	29	1(3.4%)	0.4-15.0	
Retired	28	0(0.0%)	-	
Hair Dresser	89	1(5.9%)	0.6-24.4	
Others	94	7 (7.4%)	3.4-14.1	
**Health Area**				
Mankon	267	34 (12.7%)	9.1-17.1	0.026
Alakuma	152	7(4.6%)	2.1-8.8	
Azire	106	4(3.8%)	1.3- 8.7	
Mulang	99	5(5.1%)	2.0-10.7	
Nkwen Baptist	98	5(5.1%)	2.0-10.8	
Nkwen Urban	95	4(4.2%)	1.4-9.7	
Alabukam	91	4(4.4%)	1.5-10.1	
Bamendankwe	78	3(3.8%)	1.1-9.9	
Ntankah	66	1(1.5%)	0.2-6.9	
Atuakom	42	0(0.0%)	-	
Alamandum	38	1(2.6%)	0.3-11.6	
Akumlam	38	2(5.3%)	1.1-15.8	
Nkwen Rural	19	0(0.0%)	-	
Ntamba	9	0(0.0%)	-	
Mbatchongwe	7	0(0.0%)	-	
Ndza	3	0(0.0%)	-	

## Discussion

Hepatitis B virus infection is a well-established public health problem worldwide, affecting more than 257 million people in low- and middle-income countries [4]. Massive screening and early diagnosis of HBV, as well as access to vaccination services, is the surest way to combat this growing concern, especially in endemic countries. However, only 9% of HBV-infected persons have been diagnosed worldwide [4]. Few studies have reported the population-based HBV prevalence in Cameroon, with varying results. This study, carried out in the Bamenda Health District in the North West Region of Cameroon, reported a seroprevalence of HBV at 5.8% with a significantly higher prevalence in the male subgroup, tricenarians and drivers. These findings provide important clues in directing further research and designing public health interventions to mitigate the HBV infection in Cameroon.

The reported HBV seroprevalence of 5.8% represents an intermediate endemicity [12]. It is about half (11.2%; 95% CI: 9.7 - 12.8%) of the Cameroon national prevalence found in a systematic review and meta-analysis by Bigna *et al*. [13]. Two other studies in Yaounde including 4650 and 9024 blood donors revealed a much higher seroprevalence of HBV at 12.1% and 12.6% respectively [14, 15]. Our findings could be different from what was found elsewhere in the country because of the variable socioeconomic status and inherent variations related to different geographic zones. This seroprevalence is similar to the 6.0% found in Bamenda by Abongwa *et al*. [16] and the African value (6.1%; 95% CI: 4.6 - 8.5) published by the WHO in 2017 [4]. However, our seroprevalence is higher than the 4.4% (95% CI: 1.1-9.6%) found in a cohort of 7069 pregnant women in four centres in Cameroon [17].

Males had a significantly higher seroprevalence of HBV. Hepatitis B tends to be more prevalent in men compared to women, also the condition seems to progress faster among males and they suffer the most complications. Males infected with HBV have a 6-fold likelihood of developing chronic Hepatitis [18]. This trend was also observed in Yaounde in 2013, where males were also found to have a significantly higher seroprevalence of HBV, 12.9% in males compared to 8.2% in females [15]. Our findings are also in agreement with results from Burkina Faso, where males had a significantly higher prevalence of HBV (10.5%; 95% CI: 9.6-11.4%) than women (7.8%; 95% CI: 7.1-8.6%), p = 0.0002. This observed difference in prevalence rates might reflect variations in the patterns of risk behaviours between males and females.

The higher HBV seroprevalence (8.4%; 95% CI: 5.8-11.6) observed in the 30-39 age group by this study is similar to studies done in Bamenda and Yaounde which showed the highest prevalence in the age group 31 - 40 years and 30 - 34 years respectively [15, 16]. These findings are quite unexpected, as the younger population is expected to have more risky sexual behaviour and exposure to the infection. This was the case in a study including all 10 regions of the country that found a high seroprevalence of HBV at 13.01% (95% CI: 12.03% - 14.05%) among young males 18 - 23 years [17].

The observed highest seroprevalence of HBV among drivers is not surprising. Drivers, particularly truck and long-distance bus drivers, are particularly susceptible to STIs including HBV. People in this profession spend much time outside the house and so are more likely to indulge in risky sexual activity, including sex with multiple sexual partners and commercial sex workers. In Cameroon, a study done in truck drivers revealed a high HBV prevalence of 15% [18].

**Limitations:** using a convenience sampling method from voluntary screenings is subject to selection bias, with under-representation or over-representation of particular groups within the sample. We therefore, advise that our results should be interpreted with caution especially regarding generalization of results to the study population. About half of the participants were teachers and students, therefore people from other occupations were under-represented. This is partly because the educational industry represents the primary activity in the city of Bamenda with so many primary, secondary and tertiary educational institutions. We used HBsAg as the only marker for hepatitis B infection. Other markers are available which together with HBsAg will provide more reliable results. However, our study is also strong for its large sample size and because we utilized a screening test that has been concretely validated and approved. Sending announcements wide enough through radio and churches across Bamenda, permitted us to recruit participants from a wider audience that covers the Bamenda Health District.

## Conclusion

This study found a seroprevalence of hepatitis B of 5.8%, representing an intermediate endemicity according to World Health Organization (WHO) criteria. The disease was highest amongst men, professional drivers and tricenarians. These results emphasize the need for more screening and immunization campaigns. We recommend more robust studies to further elucidate the population-based prevalence of HBV in Bamenda and Cameroon, in general, to better informed public health interventions.

### What is known about this topic

- *The prevalence of hepatitis B in Cameroon is between 6-16%;*
- *The prevalence of hepatitis B amongst blood donors in blood banks stands at 10.7-12.1%;*
- *Hepatitis B virus infection is endemic in low- and middle-income countries, especially in Africa and Asia*.

### What this study adds

- *The prevalence of hepatitis B virus in Bamenda stands at 5.8%, where males recorded the highest burden with 8.4% prevalence;*
- *The age group 30-39 years had the highest proportion of positive cases, which stands at 8.4%;*
- *Suggest a disproportionately higher burden of HBV infection amongst drivers, with a prevalence of 16.7%*.

## References

[1] World Health Organization (WHO) Hepatitis B.

[2] Center for Disease Control and Prevention Hepatitis B.

[3] Olayinka AT, Oyemakinde A, Balogun MS, Ajudua A, Nguku P, Aderinola M (2016). Se-roprevalence of Hepatitis B Infection in Nigeria: A National Survey. Am J Trop Med Hyg.

[4] World Health Organization (WHO) (2017). Global hepatitis report.

[5] Lok ASF, McMahon BJ (2007). Chronic hepatitis B. Hepatology.

[6] Frambo AAB, Atashili J, Fon PN, Ndumbe PM (2014). Prevalence of HBsAg and knowledge about hepatitis B in pregnancy in the Buea Health District, Cameroon: a cross-sectional study. BMC Res Notes.

[7] Noubiap JJN, Nansseu JRN, Ndoula ST, Bigna JJR, Jingi AM, Fokom-Domgue J (2015). Prevalence, infectivity and correlates of hepatitis B virus infection among pregnant women in a rural district of the Far North Region of Cameroon. BMC Public Health.

[8] Mbanya DN, Takam D, Ndumbe PM (2003). Serological findings amongst first-time blood donors in Yaoundé, Cameroon: is safe donation a reality or a myth?. Transfus Med Oxf Engl.

[9] Fogwe ZN (2016). An Assessment of an Urban Development-Flood-Impact Relationship in a Near Millionaire City of Cameroon (Bamenda). J Geosci Environ Prot.

[10] Hou J, Liu Z, Gu F (2005). Epidemiology and Prevention of Hepatitis B Virus Infection. Int J Med Sci.

[11] Bigna JJ, Amougou MA, Asangbeh SL, Kenne AM, Noumegni SRN, Ngo-Malabo ET (2017). Seroprevalence of hepatitis B virus infection in Cameroon: a systematic review and meta-analysis. BMJ Open.

[12] Fouelifack Ymele F, Keugoung B, Fouedjio JH, Kouam N, Mendibi S, Dongtsa Mabou J (2012). High Rates of Hepatitis B and C and HIV Infections among Blood Donors in Cameroon: A Proposed Blood Screening Algorithm for Blood Donors in Resource-Limited Settings. J Blood Transfus.

[13] Ankouane F, Noah DN, Atangana MM, Kamgaing RS, Guekam PR, Biwolé MS (2016). [Sero-prevalence of hepatitis B and C viruses, HIV-1/2 and syphilis among blood donors in the Yaoundé Central Hospital in the centre region of Cameroon]. Transfus Clin Biol J Soc Francaise Transfus Sang.

[14] Ntonifor H (2015). Sero-Prevalence of Human Immuno deficiency virus HIV and Hepatitis B virus Co-infection in pregnant women in Bamenda. Int J Curr Microbiol Appl Sci.

[15] Dionne-Odom J, Mbah R, Rembert NJ, Tancho S, Halle-Ekane GE, Enah C (2016). Hepatitis B, HIV, and Syphilis Seroprevalence in Pregnant Women and Blood Donors in Cameroon. Infect Dis Obstet Gynecol.

[16] Yang F, Yin Y, Wang F, Zhang L, Wang Y, Sun S (2010). An Altered Pattern of Liver Apolipoprotein A-I Isoforms Is Implicated in Male Chronic Hepatitis B Progression. J Proteome Res.

[17] Noah DN, Andoulo FA, Essoh BVM, Ayissi GN, Bagnaka SAFE, Sida MB (2015). Prevalence of HBs Antigen, and HCV and HIV Antibodies in a Young Male Population in Cameroon. Open J Gastroenterol.

[18] HIV and STD prevalence among bus and truck drivers in Cameroon (1994). AIDS Anal Afr.

